# Vision-Threatening Juvenile Lupus Retinopathy: A Report of Two Cases and Review of the Literature

**DOI:** 10.31138/mjr.220625.rra

**Published:** 2026-03-01

**Authors:** Kaustav Bhowmick, Somnath Chakraborty, Sonali Dey, Saptadeep Misra, Juber Khan, Arghya Chattopadhyay, Pasang Lhamu Sherpa

**Affiliations:** 1 Department of Internal Medicine, North Bengal Medical College and Hospital, West Bengal, India;; 2 Retina Institute of Bengal, West Bengal, India;; 3 Department of Clinical Immunology and Rheumatology, Institute of Post-Graduate Medical Education and Research, Kolkata, India;; 4 Department of Medicine, North Bengal Medical College and Hospital, West Bengal, India;; 5 Department of Rheumatology, North Bengal Medical College and Hospital, West Bengal, India

**Keywords:** paediatrics, lupus erythematosus, systemic, retinal diseases

## Abstract

**Aim:**

The present case-based review aims to highlight two successfully treated cases of juvenile lupus retinopathy in a 15-year-old and a 17-year-old female with a review of literature highlighting the clinical profile and the treatment modalities used in those cases.

**Methods:**

A comprehensive literature search for vision-threatening juvenile lupus retinopathy on Medline/PubMed, Scopus, Web of Science, and Directory of Open Access Journals (DOAJ) revealed 27 cases from 25 case reports.

**Results:**

The interval between visual loss and treatment initiation was three days (range: 0- 2 months). The most prevalent fundoscopic findings were cotton-wool spots in 18 (66.7%). IV Cyclophosphamide (CYC) was the most commonly used immunosuppressive agent in 12 cases (44.4%). Retinal photocoagulation was the principal intraocular treatment modality employed in 11 cases (40.7%). The majority of JLR cases (17; 63%) showed excellent vision recovery.

**Conclusion:**

Juvenile lupus retinopathy is a rare yet severe manifestation of juvenile lupus and can have excellent outcomes if addressed early and aggressively.

## INTRODUCTION

Ocular involvement occurs in approximately one-third of cases of systemic lupus erythematosus (SLE),^1^ with lupus retinopathy being the second most common ocular manifestation, following dry eyes.^2.^ Lupus retinopathy affects 12% of SLE patients, ranging from mild to severe blinding forms.^3^ Childhood-onset SLE, which constitutes 15–20% of SLE cases, often results in a more aggressive disease course and higher incidences of major organ involvement compared to their adult counterparts.^4^ The prevalence of lupus retinopathy is well-documented in adults but scarcely reported in children. Retinopathy in juvenile lupus typically arises from immune-complex-mediated microangiopathy, followed by vaso-occlusive retinopathy and retinal vasculitis.^3^ This article highlights two interesting cases of vision-threatening juvenile lupus retinopathy, which were successfully treated with aggressive systemic immunosuppression. In one case, intravitreal triamcinolone acetonide (IVTA) was also administered. We reviewed case reports and case series on vision-threatening lupus retinopathy, focusing on demographics, extraocular clinical domains, autoantibodies with special mention of antiphospholipid antibodies (APLA), serological disease activity, treatment, and visual outcomes. We also analysed findings from fundoscopy, optical coherence tomography (OCT), and fundus fluorescein angiography (FFA), comparing them with our cases.

## PRESENTATION OF THE CASES

### Case 1

A 17-year-old girl from North-Eastern India was admitted in July 2024 with a sudden, painless loss of vision in both eyes lasting over two weeks. Her symptoms began in January 2023, when she experienced a low-grade fever, photosensitive malar rash, polyarthralgia, painless palatal ulcer, and an erythematous, painful, maculopapular rash over her palms and soles lasting about a month before she sought medical consultation. Her evaluation revealed a haemoglobin (Hb) of 11.6 g/ dl, leukopenia with a total leucocyte count (TLC) of 3.7 X 10^3^ /μL, lymphopenia with an absolute lymphocyte count of 370/μL, and mild thrombocytopenia with a platelet count of 110 X 10^3^ /μL. At that time, her urinalysis reports, renal function tests, and liver function tests were within normal limits. She had an elevated serum high-sensitivity C-reactive protein (hs-CRP) value at 1.4 mg/dl (<0.3 mg/dL). Immunological workup revealed antinuclear antibody (ANA) positivity in the Hep 2010 cell line by indirect immunofluorescence (IIF) with a 3+ homogenous pattern at 1:160 dilution. Extractable nuclear antigen (ENA) blot showed positivity for several autoantibodies, with an intensity of 3+ for Smith (Sm), 3+ for anti-double-stranded deoxyribonucleic acid (anti-dsDNA), 2+ for histone (Hi), 2+ for nucleosome (Nuc) 2+, and 3+ for ribosomal P (Ribo P). She also had low serum complements, with a serum C3 level of 72 mg/dl (90 – 180 mg/dl) and a C4 level of 9 mg/dl (10–40 mg/ dl). She fulfilled the 2019 European League Against Rheumatism/American College of Rheumatology (EULAR/ACR) classification criteria for SLE,^5^ with a score of 21 (cut-off of 10). Given vasculitic rashes over the palms and soles she was initiated on prednisolone 40 mg/d (1mg/kg/d), and hydroxychloroquine (HCQ). Her prednisolone dose was tapered down over 3 months to 5 mg/d while HCQ was continued. No steroid-sparing agent was used during this time.

**Figure 1.(above).**
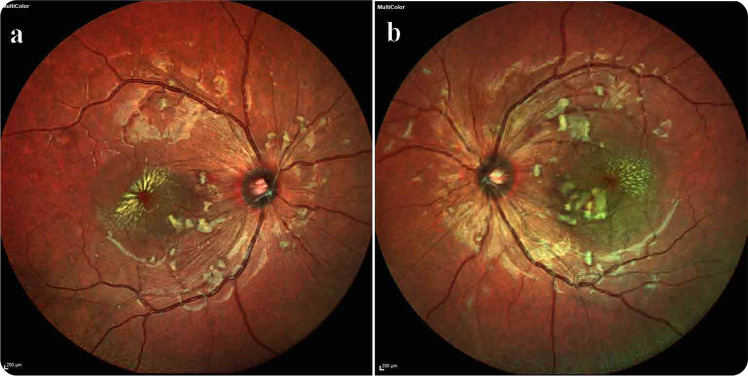
Multicolour fundus photograph showing macular oedema with perifoveal hard exudates arranged in a fan-shaped pattern and multiple cotton wool spots in both eyes (a, b), along with a perifoveal flame-shaped bleed inferonasal to the fovea in the left eye b).

**Figure 2.(below).**
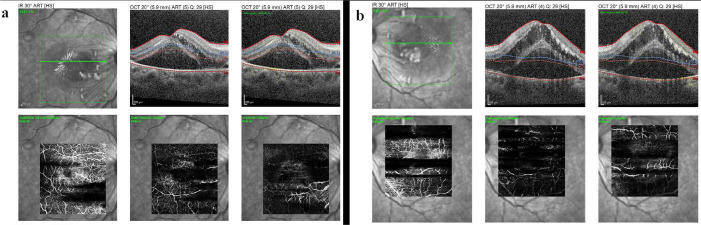
Optical coherence tomography (OCT) reveals serous detachment with intra-retinal fluid nasally and hyper-reflective spots corresponding to the hard exudates in the right eye a), and intra-retinal fluid all across with hyper-reflective spots corresponding to the hard exudates in the left eye b).

**Figure 3.(above).**
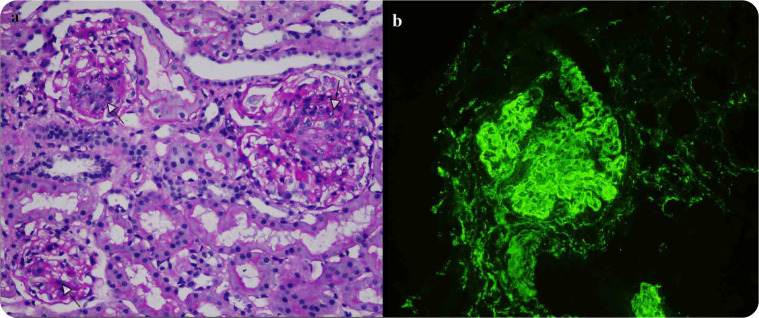
Renal biopsy specimen stained with Periodic Acid Schiff stain (PAS) a) showing glomeruli with endocapillary hypercellularity and neutrophilic infiltration (arrows) (PAS, × 400), and granular C1q positivity present over the mesangium and capillaries on immunofluorescence b) (immunofluorescence for C1q, anti-human-C1q antibodies marked with fluorescein, fluorescence microscopy, × 400).

**Figure 4.(below).**
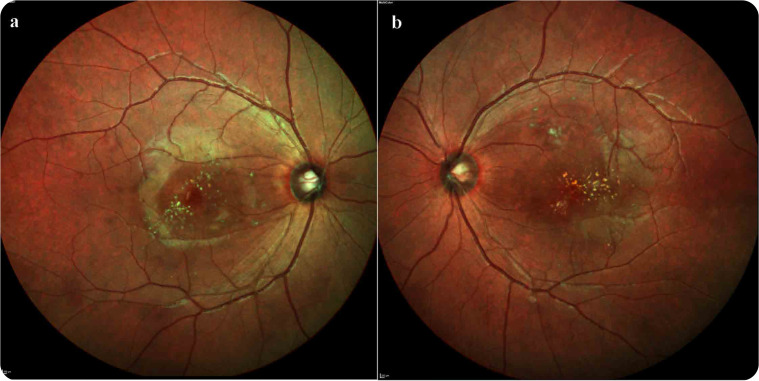
Multicolour fundus photograph after eight months showing resolved macular oedema with few residual hard exudates in both eyes. There are no residual cotton wool spots in the right eye a), and a few resolving cotton wool spots superior to the fovea in the left eye b).

**Figure 5.(above).**
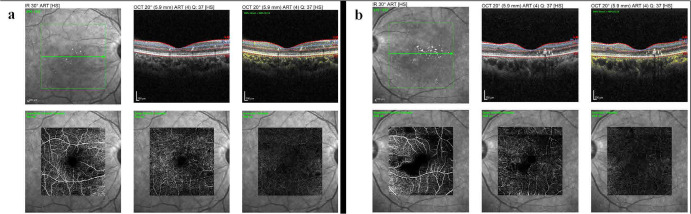
Repeat OCT scans after eight months showing resolved serous detachment, resolved intra-retinal fluid, and few hyper-reflective dots with after shadow suggestive of residual hard exudates in the right eye a), and a similar picture in the left eye b), except for some residual intra-retinal fluid nasal to fovea.

She was admitted yet again with sudden painless loss of vision. Over the past 2 weeks, she also experienced an intermittent low-grade fever, peaking at 100.2°F. It coincided with the appearance of malar rash, and alopecia. Neurological signs and symptoms were absent and her best corrected visual acuity (BCVA) was 3/60 in both eyes. Blood pressure was 140/90 mmHg in the left arm. Fundus examination showed macular oedema, perifoveal hard exudates in a fan-shaped pattern, multiple cotton wool spots in both eyes and a perifoveal flame-shaped bleed inferonasal to the fovea in the left eye (**Figure 1A–B**). Optical coherence tomography (OCT) of the right eye revealed serous detachment with intra-retinal fluid nasally and hyper-reflective spots corresponding to the hard exudates, while the left eye OCT demonstrated intra-retinal fluid in all quadrants along with similar hyper-reflective spots as in the right (**Figure 2A–B**). The optical coherence tomography angiography (OCTA) (**Figure 2A–B**) image indicated a segmentation error, due to significant serous detachment making it impossible to comment on the vascular complexes. Laboratory tests showed Hb of 9.2 g/dl, TLC of 9 X 10^3^ /μL, and platelet count of 121 X 10^3^ /μL. Liver function tests were within normal range. Serum hs-CRP levels were slightly raised at 0.5 mg/dl (<0.3 mg/ dl). A magnetic resonance imaging scan (MRI) of the brain, done to rule out cerebral lupus, was unremarkable. Infectious disease workup was negative for Interferon-gamma release assays (IGRA), syphilis, toxoplasma, toxocara, and Cytomegalovirus (CMV) serologies. Urinalysis showed active sediments with a protein ‘+’, white blood cells (WBCs) 10–12/high power field (hpf), and red blood cell (RBC) casts 4–5/hpf, with no bacterial growth found on urine culture. 24-hour-urine protein revealed proteinuria of 532 mg with a serum creatinine of 0.7 mg/dl. Her serum complements were low with a serum C3 of 52 mg/dl and a serum C4 of 7 mg/dl. She also had high anti-dsDNA titres at 600 IU/ml on the ELISA test. She tested negative for anti-cardiolipin (aCL) IgG and IgM antibody, anti-β2 glycoprotein I (anti-β2 GPI) IgG and IgM antibody, and lupus anticoagulant. Based on these findings she was diagnosed with lupus retinopathy and lupus nephritis. Renal biopsy was suggestive of Class IV lupus nephritis (**Figure 3**) with an activity score of 7/24 and a chronicity score of 0/12 as per the National Institute of Health (NIH) modification of the International Society of Nephrology/Renal Pathological Society (ISN/RPS) classification of lupus nephritis.^6^ Her Safety of Estrogens in Lupus Erythematosus National Assessment – Systemic Lupus Erythematosus Disease Activity Index (SELENA-SLEDAI) ^7^ score was 25 suggestive of a severe disease. She was administered intravenous methylprednisolone (IV MP) 500 mg for three days followed by oral prednisolone at 0.75 mg/kg/d. She received six monthly doses of intravenous cyclophosphamide (IV CYC) as per the modified NIH protocol for the treatment of lupus nephritis.^8^ She is currently being maintained on mycophenolate mofetil (MMF) at a dosage of 1.5 g/d. Her glucocorticoid dose has been tapered to 6.25 mg/d after eight months, and she has consistently taken hydroxychloroquine (HCQ) at 5 mg/kg/d. After eight months, her BCVA improved to 6/9 in both eyes. Repeat fundus images showed resolved macular oedema with a few residual hard exudates in both eyes. There are no cotton wool spots in her right eye, and a few resolving ones are superior to the left eye’s fovea (**Figure 4A–B**). Repeat OCT scans reveal no serous detachment and intra-retinal fluid, but a few hyper-reflective dots with after shadow suggestive of residual hard exudates in the right eye, and a similar picture in the left eye, except for some residual intra-retinal fluid nasal to fovea (**Figure 5A–B**).

### Case 2

**Figure 6.**
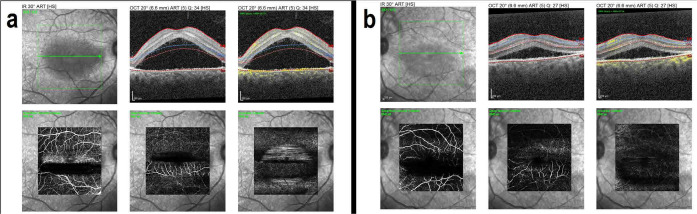
(below). Optical coherence tomography (OCT) shows serous retinal detachments in both eyes with the right eye a) involved more than the left eye b).

**Figure 7.**
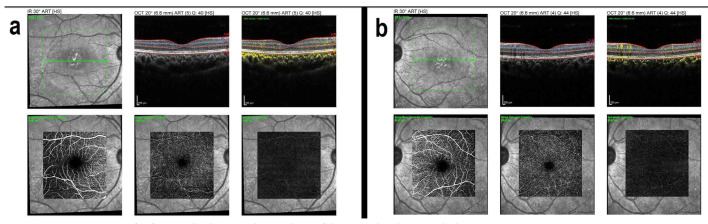
Repeat OCT scans after four months show complete resolution of the serous detachments in both eyes.

A 15-year-old girl from Eastern India was admitted with a progressive, painless loss of vision in both eyes over the past month. Two months earlier, she had developed symmetric, inflammatory polyarthritis involving the small joints of hands and feet, along with photosensitive malar rashes, discoid lupus erythematosus (DLE) rashes over her face and ears, painless palatal ulcers, and non-scarring alopecia. Upon admission, her visual acuity was limited to counting fingers (FC) at one meter in both eyes. She was hemodynamically stable, with a blood pressure of 120/70 mm Hg, and had a mild non-tender splenomegaly on clinical examination. Fundus examination showed macular oedema, extensive cotton wool spots, and some retinal haemorrhages. OCT showed serous retinal detachments in both eyes, with the right eye being more affected. OCTA image showed segmentation error, due to significant serous detachment (**Figure 6A–B**). Laboratory tests showed Hb of 6.5 g/dl, TLC of 3.2 X 10^3^ /μL, an absolute lymphocyte count of 800 /μL, and a platelet count of 180 X 10^3^ /μL. The mean corpuscular volume (MCV) was 102 fl with peripheral blood smear showing anisopoikilocytosis and polychromasia. The reticulocyte production index was 2.6, and she tested positive for direct Coombs’ test. The evaluation for vitamin B12 deficiency and hemoglobinopathy was negative. However, her ferrokinetics showed 16% transferrin saturation, indicating concurrent iron-deficiency anaemia and a serum ferritin value of 1600 μg/L. She had indirect hyperbilirubinemia with a raised SGOT of 80 IU/L and a normal SGPT of 23 IU/L. The serum hs-CRP level was 0.4 mg/dL. A brain MRI done to rule out cerebral lupus was within normal limits. She was screened negative for IGRA, syphilis, toxocara, and CMV serologies. Urinalysis showed active sediments with protein ‘+’, and WBCs 5–6/hpf, but no bacterial growth on culture. She had sub-nephrotic range proteinuria with a 24-hour urine protein value of 778 mg and a normal renal function. ANA in the Hep 2010 cell line by IIF at 1:160 dilution was positive and exhibited a 4+ coarse speckled pattern. ENA blot showed positive autoantibodies: U1 ribonucleoprotein (U1-RNP) 1+, Sjogren’s syndrome-related antigen-A (SS-A) 2+, Ro-52 2+, Sjogren’s syndrome-related antigen-B (SS-B) 1+, Nuc 1+, and Sm 1+. She had low serum complements with a serum C3 of 23 mg/dl, serum C4 of 4 mg/dl, and a high anti-dsDNA titre of 452 IU/ml. She tested negative for antiphospholipid antibodies (APLA). Based on the 2019 EULAR/ ACR classification criteria for SLE,^5^ she was diagnosed with lupus with a total score of 26. Her clinical components were serous retinopathy secondary to lupus, autoimmune haemolytic anaemia (AIHA), nephritis, leukopenia, mucocutaneous symptoms, and arthritis. Renal biopsy was suggestive of a Class III lupus nephritis with an activity score of 3/24 and a chronicity score of 0/12 as per the NIH modification of the ISN/RPS classification of lupus nephritis.^6^ Her SELENA-SLEDAI^7^ score also stood at 25, suggestive of s severe disease. She received IV MP 500 mg for 5 days, followed by oral prednisolone (0.75 mg/kg/d). IVCYC, as per the modified NIH protocol for lupus nephritis^8^ was initiated as the steroid-sparing agent. However, even after 10 days of starting treatment, there was no improvement in her visual acuity, which remained at FC of 2m in both eyes. She was administered two doses of intravitreal triamcinolone (IVTA) on two consecutive days by the vitreoretinal surgeon. By the end of 1 month, she had a remarkable improvement, and her BCVA was 6/12 in the right eye and 6/24 in the left eye. Following four monthly doses of IV CYC, her BCVA improved to 6/9 in the right eye and 6/12 in the left. Her steroid dose was tapered to 10 mg/d of prednisolone. She remained on HCQ throughout the entire course of her treatment. Repeat OCT scans after four months showed complete resolution of the serous detachments (**Figure 7A–B**). She was further planned for MMF as her maintenance therapy.

A written informed consent was obtained from the patients for the publication of this case-based review and the accompanying images.

## COMPREHENSIVE SEARCH STRATEGY

We extensively searched Medline/PubMed, Scopus, Web of Science, and Directory of Open Access Journals (DOAJ) for existing literature on vision-threatening juvenile lupus retinopathy (JLR) till June 2025. Juvenile SLE (JSLE) was defined as patients diagnosed with lupus under the age of 18.^9^ Lupus retinopathy includes microangiopathy, vaso-occlusive retinopathy, and retinal vasculitis.^10^ The Medical Subject Headings (MeSH) keywords “Pediatrics”, “Lupus Erythematosus, Systemic”, AND “Retinal Diseases” were combined as a part of the search strategy. We did not impose any time restrictions on publication dates, nor did we limit our search by language. All articles, including titles, abstracts, and full texts, were independently reviewed by three authors (KB, SM, SD).

During the literature search, 35 articles were identified, with 25 deemed eligible for the current study.^11–35^ Excluded articles included those on JLR with preserved visual acuity,^36^ cohort studies without detailed discussion on the individual cases,^37^ hypertensive retinopathy in JSLE,^38^ haemorrhagic retinopathy secondary to thrombocytopenia in JSLE,^39^ JSLE with neuro retinitis,^40^ optic neuropathy,^41,42^ coexistent choroidopathy in JSLE,^43,44^ and retinopathy secondary to the effects of drugs like hydroxychloroquine in JSLE.^45^

Parameters regarding clinical manifestations and treatment outcomes were recorded. For data analysis, IBM SPSS V21.0 was used.

**Figure 8.**
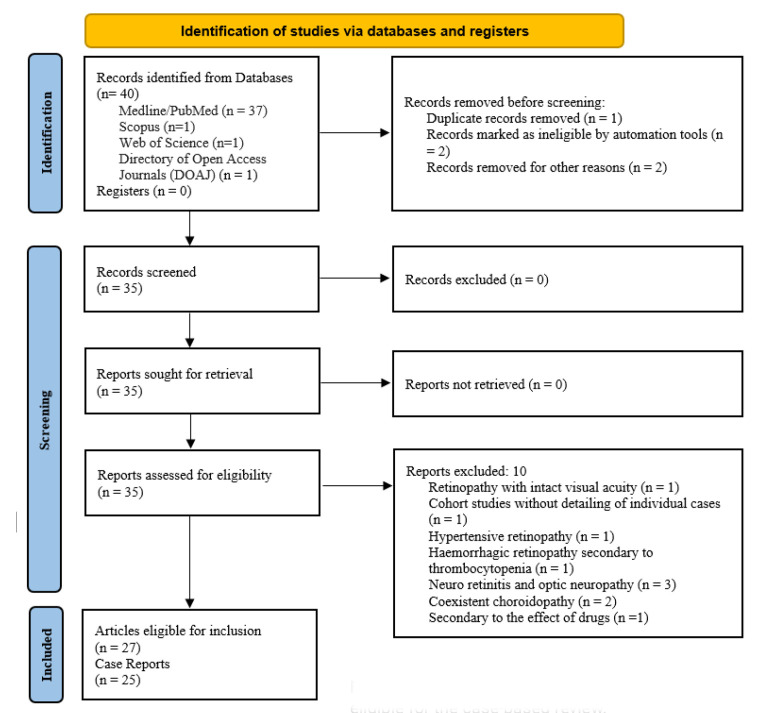
Flow chart showing selection of the studies eligible for the case-based review.

**Figure 8** elucidates the flow chart of the selection of the eligible studies for the case-based review.

## LITERATURE REVIEW

A literature search identified 27 cases of juvenile lupus retinopathy from 25 case reports. Clinical characteristics, immunological parameters, and treatments of these patients, along with our present cases, are summarised in **Table 1**, while findings from fundoscopy, OCT, and FFA, where available, along with the visual outcomes, are summarised in Table 2. The median duration of lupus before the onset of this condition was one month (range: 0- 60 months). The interval between visual loss and treatment initiation was three days (range: 0- 2 months). Among the 19 cases with available APLA test results, six (31.6%) tested positive. The most prevalent fundoscopic findings were cotton-wool spots in 18 (66.7%) and intraretinal haemorrhages in 16 (59.3%) cases. Prompt and aggressive systemic immunosuppression was the cornerstone of therapy, with IV CYC being the most commonly used immunosuppressive agent in 12 cases (44.4%). Intraocular treatment was essential in 13 cases (48.1%), with retinal photocoagulation being the predominant modality employed in 11 cases (40.7%).

**Table 1.** Clinical characteristics and various treatment modalities of juvenile lupus retinopathy patients.

**Author (Year)**	**Age/Gender**	**Duration of SLE**	**Laterality**	**Active clinical domains**	**Autoantibodies**	**APLA profile**	**C3**	**C4**	**HCQ**	**Time from LOV to treatment initiation**	**IST**
Silvermann et al. (1978)^11^	15 Y/M	3 months	LE	Malar rash Polyarthritis Oral ulcers	dsDNA	NA	Low	NA	NA	3 days	PDN
Vine et al. (1984)^12^	14 Y/M	4 years	B/L	Malar rash Polyarthritis Oral Ulcers	NA	NA	Normal	Normal	NA	NA	PDN, Laser Photocoagulation
											
Ho et al. (2008)^13^	16 Y/F	Simultaneous	B/L	Malar rash, oral ulcers, arthritis, leukopenia	ANA, dsDNA	Neg	Low	Low	Yes	1 week	IVMP, PRP, cryoretinopexy (RE), intravitreal t-PA (LE)
RT Almeida et al. (2011)^14^	15Y/F	1 month	B/L	Malar rash, Pericarditis, NPLE, class III LN, Cutaneous vasculitis, polyserositis,	dsDNA	Neg	Low	Low	NA	NA	IV MP, PDN, CYC six monthly pulses 1g/m^2^, AZA
Donnithorne et al. (2013)^15^	16Y/F	10 months	LE	Constitutional.	SS-A, SS-B, RNP, and dsDNA	Neg	NA	NA	Yes	NA	IV MP, RTX 2 induction doses CYC monthly pulses, PRP
Donnithorne et al. (2013)^15^	13Y/F	1 month	RE	Constitutional, Polyarthritis	Sm, dsDNA, RNP, and SS-A	Neg	NA	NA	Yes	NA	IV MP, RTX 2 weekly induction doses, CYC seven monthly pulses, MMF 1 g/d maintenance
Rony Gelman et al. (2014)^16^	12Y/F	15 months	B/L	Malar rash, NPLE, constitutional,	SS-A, SS-B, Sm, RNP, ribosomal-P	Neg	Low	Low	NA	Immediate	RTX, Peripheral scatter laser photocoagulation.
Satoshi Sato et al. (2015)^17^	11Y/F	11 months	B/L	NPLE	dsDNA	Neg	Normal	Normal	NA	Immediate	IV MP, IVIG, IV CYC six monthly pulses, six PLEX sessions
Parchand et al. (2017)^18^	16 Y/F	6 months	LE	Constitutional, arthritis, alopecia, cervical adenopathy, palatal ulcers, leukopenia, lymphopenia.	ANA	Neg	NA	Low	NA	1 day	High-dose steroids, MTX, anticoagulants, PRP
Y. Kotani et al. (2018)^19^	14Y/F	Simultaneous	LE	Constitutional, Mucocutaneous, Class II LN,	dsDNA,	aCL	Low	Low	NA	NA	IV MP, Oral PDN, Tac, IV Heparin, Aspirin
Palkar et al. (2018)^20^	15 Y/F	18 days	RE	Constitutional, Skin rashes, arthralgia, myalgia	ANA, dsDNA	Neg	NA	NA	Yes	18 days	Oral PDN 1mg/kg
Mori et al. (2019)^21^	16 Y/M	4 months	B/L	Constitutional, malar rash, arthritis, alopecia, leukopenia	U1-RNP, SS-A	aCL	Low	Low	No	NA	IV MP, PDN, IV CYC 750mg/m^2^body surface area, RTX 375 mg/m^2^weekly × 4 doses, Aspirin, AZA
Moreno Paramo et al. (2019)^22^	14 Y/M	Simultaneous	LE	Constitutional, pleuritis, LN, thrombocytopenia, hepatomegaly	ANA	aCL, LA	NA	NA	NA	36 hours	Steroids, anticoagulation, anti-VEGF, pars plana vitrectomy
Huang et al. (2020)^23^	11Y/F	1 month	B/L	LN, rash, polyserositis	dsDNA, Nuc,	aCL	Low	Low	NA	24 hours	IV MP Laser PRP IV Dexa, Low dose MP, MMF
Somya Ish et al. (2020)^24^	17Y/M	1 year	B/L	DAH	dsDNA	NA	Low	Low	NA	NA	IV MP, CYC three monthly pulses 750mg, RTX 4 weekly induction doses, MMF maintenance
Firl et al. (2020)^25^	12 Y/F	5 years	B/L	Maculopapular rashes face and extremities Alopecia Constitutional	dsDNA	NA	NA	Low	Yes	2 weeks	Anti-VEGF IV MP, Oral PDN, RTX
Guleria et al. (2020)^26^	9Y/M	Simultaneous	B/L	Constitutional, pancytopenia, NPLE.	dsDNA	Neg	Normal	Normal	Yes	2 weeks	IV MP, Oral PDN, warfarin, aspirin, CYC six monthly pulses 500 mg/m^2^, AZA 2 mg/kg/d maintenance
Deaner et al. (2020)^27^	15 Y/M	1 year	B/L	Malar rash	dsDNA, Sm, RNP, SS-A	aCL IgM, β2GP IgM	NA	Low	NA	2 weeks	IV MP, Oral PDN, LMWH 1 mg/kg SC twice daily, RTX 1 g every 2 weeks, MMF 3g/d,
Bretas et al. (2021)^28^	13 Y/F	Simultaneous	B/L	Non-haemolytic anaemia, thrombocytopenia, serositis, LN	ANA	Neg	NA	NA	NA	Immediate	IVIG, IV MP, Oral PDN, CYC
Alhassan et al. (2021) ^29^	14Y/F	Simultaneous	B/L	Arthralgias, Constitutional	dsDNA	NA	Normal	Normal	Withheld	Immediate	IV MP, PDN, AZA,
Rashmi Roongta et al. (2021)^30^	15Y/M	2 weeks	RE	Pancytopenia, AIHA, Cutaneous SVV	dsDNA, SS-A	Neg	Normal	Normal	NA	2 weeks	IV MP, Oral PDN, CYC six monthly pulses 750 mg
Jin Hwan Jeon et al. (2022)^31^	13Y/F	2 months	B/L	Constitutional, Pancytopenia, AIHA	dsDNA, Sm, RNP	LAC, anti-β2GPI	Low	NA	Yes	NA	IV MP, IVTA, IV Dexa implant, PRP
Ahmad Zeeshan Jamil et al. (2022) ^32^	15Y/F	6 months	B/L	Constitutional, Malar rash, Non-scarring Alopecia, Cutaneous SVV, Pancytopenia	NA	NA	Low	Low	Yes	2 days	IV MP, Oral PDN, AZA 50 mg/d
Parakh et al. (2023)^33^	9Y/F	Simultaneous	B/L	Arthralgia ACLE Constitutional Leukopenia	Sm, SS-A, SS-B, RNP	NA	Low	NA	Yes	15 days	IV MP, PDN, CYC six monthly pulses 450mg, AZA, Laser photocoagulation LE
Babu Kalpana et al. (2023)^34^	17 Y/F	NA	LE	Class IV LN	NA	Neg	Low	Low	NA	2 months	IV MP, PDN, CYC pulses 500 mg 2 weekly 6 doses, Laser photocoagulation
Sharum et al. (2024)^35^	12Y/F	1 month	RE	Constitutional, pancytopenia, AIHA	Ro	Neg	NA	NA	NA	NA	IV MP, Oral PDN, laser PRP IV CYC
Sharum et al. (2024)^35^	12Y/ M	NA	B/L	Cerebral Lupus	NA	NA	NA	NA	NA	1 month	PRP, PDN, AZA
Current (2025)	17 Y/F	18 months	B/L	Nephritis Malar rash Alopecia Constitutional	Sm, Hi, Nuc, Ribo-P, dsDNA	Neg	Low	Low	Yes		IV MP, PDN, IV CYC 6 monthly doses, MMF
Current study (2025)	15 Y/F	2 months	B/L	Nephritis AIHA, leukopenia Arthritis Malar rash DLE Alopecia	U1RNP, Nuc, Sm, SS-A, SS-B, Ro-52	Neg	Low	Low	Yes		IV MP, PDN, IVTA, IV CYC monthly doses

aCL: anti-Cardiolipin Antibody; ANA: Antinuclear antibody; AZA: Azathioprine; ACLE: Acute cutaneous lupus erythematosus; AIHA: Autoimmune haemolytic anaemia; APLA: Antiphospholipid antibody; B/L: Bilateral; β2GPI: Beta2 glycoprotein I; CYC: Cyclophosphamide; DAH: Diffuse alveolar haemorrhage; Dexa: Dexamethasone; dsDNA: double-stranded deoxyribonucleic acid; F: Female; HCQ: Hydroxychloroquine; IST: Immunosuppressive therapy; IV: Intravenous; IV Dexa: Intravitreal dexamethasone; IVIG: Intravenous Immunoglobulin; IVTA; Intravitreal triamcinolone acetonide; LE: Left Eye; LN: Lupus Nephritis; LAC: Lupus anticoagulant; LOV: Loss of vision; M: Male; MMF: Mycophenolate mofetil; MP: Methylprednisolone; MTX: Methotrexate; NA: Not Applicable; NPLE: Neuropsychiatric Lupus erythematosus; Nuc: Nucleosome; PDN: Prednisolone; PLEX: Plasmapheresis; PRP: Panretinal Photocoagulation; RE: Right Eye; RTX: Rituximab; SS-A: Sjögren's-syndrome-related antigen A; SS-B: Sjögren's-syndrome-related antigen B; Sm: Smith; SLE: Systemic lupus erythematosus; SVV: Small vessel vasculitis; Tac: tacrolimus; t-PA; tissue plasminogen activator; U1RNP: U1 Ribonucleoprotein; VA: Visual Acuity; VEGF: Vascular endothelial growth factor.

**Table 2.** Baseline visual acuity, fundoscopic, OCT, FFA findings, and the visual outcomes after a follow-up period.

**Author (Year)**	**BCV**_A_ **(Baseline)**		**Fundoscopic finding (baseline)**	**OCT finding (baseline)**	**FFA finding (baseline)**	**Type of retinopathy**	**Follow-up period**	**BCV**_A_ **(Follow-up)**		**Outcome**
	**OD**	**OS**						**OD**	**OS**	
Silvermann et al. (1978)^11^	20/20	PL	OS: IRH, VD, narrowed arterioles.	NA	NA	CRVO and CRAO vaso-occlusive retinopathy	2 weeks	20/20	PL	No improvement of vision, large VH
Vine et al. (1984)^12^	20/20	20/20	OU: CWS in macula, Occluded retinal venules and arterioles perimacular, Scattered IRH	NA	OU: Complete occlusion of major retinal venules and arterioles. Late fluorescein angiogram extensive dye leakage	Retinal vasculitis complicated by neovascularization and VH	17 months	20/25	20/50	Deterioration of vision
Ho et al. (2008)^13^	FC 30 cm	6/60	Extensive CWS, predominantly posterior pole	NA	OD: complete obliteration of the macular capillary and widespread occlusion in the smaller vessels, OS: similar picture with partial sparing of the vascular network around the central macula	Retinal vasculitis	2 years	FC 10 cm	6/10	OD: Blind. OS: Improved
RT Almeida et al. (2011)^14^	20/200	20/200	Severe retinal vasculitis with hemorrhage	NA	NA	Retinal vasculitis	6 months	20/200	20/200	No improvement ( Legally blind)
Donnithorne et al. (2013)^15^	20/20	FC 2 ft.	OU: Macular ischemia, NVR VH Tractional RD	NA	NA	BRAO, NAION	17 months	20/20	20/200	Improved
Donnithorne et al. (2013)^15^	20/80	20/20	OD: Multiple CWS IRH BRAO	NA	NA	Retinal vasculitis with BRAO	7 months	20/30	20/20	Near complete recovery
Rony Gelman et al. (2014)^16^	20/800	20/800	OU: IRH. OS: foveal RH	OD: Nasal macular oedema OS: Diffuse macular oedema involving the fovea.	Profound vascular nonperfusion with severe macular and peripheral ischemia	Vaso-occlusive retinopathy complicated by VH	48 months	20/125	20/200	Improvement
Satoshi Sato et al. (2015)^17^	0.1	0.9	Multiple CWS	Macular oedema	NA	Retinal vasculitis	6 months	1.5	1.5	Complete recovery
Parchand et al. (2017)^18^	6/6	PL	OD: CWS, OS: Disc oedema, Dilated and tortuously thrombosed retinal veins, Arteriolar attenuation, Extensive flame-shaped IRH, Retinal pallor CRS macula	NA	NA	Vaso-occlusive retinopathy (CRAO and CRVO)	NA	6/6	PL	OS: No improvement Vitreous haemorrhage.
Y. Kotani et al. (2018)^19^	1.5	0.09	OS: Retinal arteriovenous vasculitis. Soft Vitiligo.	OS: Marked macular oedema.	OS: Retinal arteriovenous vasculitis, strong ischemia in the macular region, no reflux area, and leakage from the blood vessel	OS: Retinal vasculitis	2 months	1.5	1.2	OS; Improved vision.
Palkar et al. (2018)^20^	20/200	20/20	OD: Circumscribed area of retinal whitening, abutting the fovea nasally and inferonasal with ODE, Marked venous tortuosity, few IRH, CWS	NA	OD: Early and late hypofluorescence corresponding to the area of retinal whitening along with late disc staining	OD: Retinal vasculitis	4 weeks	20/40	20/20	OD: Marked improvement
Mori et al. (2019)^21^	0.8	0.4	Bilateral CWS and RAO.	NA	OU: Areas of retinal ischemia.	Retinal vasculitis and vaso-occlusive retinopathy	1 month	1.0	1.2	OU: Marked improvement
Moreno Paramo et al. (2019)^22^	6/6	PL	Generalised retinal paleness, ODE, VD and tortuosity, flame and spot IRH in the 4 quadrants, pre-RH in the temporal sector, CRS	NA	OU: Significant delay in arterial filling and generalized hypofluorescense due to filling defect.	Vaso-occlusive retinopathy (CRAO and CRVO)	12 months	6/6	PL	OS: No improvement (Legally blind) With VH
Huang et al. (2020)^23^	20/20	HM 10 cm	OS: CRAO and CRVO, Diffuse RH, Tortuous VD, ODE	OU: Severe macular oedema and uplifted. Massive effusion between the outer layers of the neuroretina.	NA	Retinal vasculitis and vaso-occlusive retinopathy	4 months	6/6	20/125	OS: Improved
Somya Ish et al. (2020)^24^	6/60	6/36	OD: Media hazy, Coalescent hypopigmented lesions with fluffy margins superotemporal to disc, Macular oedema. OU: Similar lesions in inferotemporal quadrant withmacular oedema	NA	OD: corresponding frank leakage. OU: frank leak	Retinal vasculitis	4 months	FC at 4m	6/24	OD: Deterioration of vision OU: Improved vision.
Firl et al. (2020)^25^	20/25	20/25	Diffuse IRH in the posterior pole and temporal NVR, (worse in OD), Macular traction and striae, Extensive peripheral VS.	NA	Temporal hyperfluorescence, Neovascularization Diffuse peripheral nonperfusion	Retinal vasculitis	1 week	20/40	20/30	Stable
Guleria et al. (2020)^26^	PL 3 ft.	PL 3 ft.	OU: CWS	OU: Central retinal atrophy.	OU: Capillary ‘drop-out’, Vessel-wall staining and leakage of dye, Significant central non-perfusion of the retina	Retinal vasculitis	12 months	6/36	6/36	OU: Improved
Deaner et al. (2020)^27^	20/20	20/80	OU: Multiple clustered CWS in the macula and posterior pole, Segmental VS peripheral retina	OU: Inner retinal hyperreflectivity and thickening, CME, Subretinal fluid.	OU: Marked macular capillary non-perfusion, arteriole occlusions, delayed venous filling, late macular leakage, segmental peripheral vascular leakage.	Vaso-occlusive retinal vasculitis	20 months	20/20	20/20	OU: Complete recovery
Bretas et al. (2021)^28^	HM	HM	OU: CWS, diffuse IRH, VS	NA	NA	Frosted branch angiitis	4 months	20/50	20/200	OU: Improvement
Alhassan et al. (2021)^29^	Blurry	More Blurred	OU: Diffuse IRH, White retinal lesions, ODE	NA	NA	Panuveitis, severe bilateral retinal vasculitis	1 year	NA	NA	OU: Significant improvement
Rashmi Roongta et al. (2021)^30^	6/60	6/6	OD: VS of the central retinal vessels, CWS, Dot IRH	NA	NA	Retinal vasculitis	6 months	6/9	6/6	OD: Marked improvement
Jin Hwan Jeon et al. (2022)^31^	HM	15/20	OU: exudate at optic disc, oedematous macula, VS, haemorrhage around the ghost vessels.	Massive exudation at both eyes with macular oedema was noted.	NA	Retinal vasculitis	NA	NA	NA	OU: Improved
Ahmad Zeeshan Jamil et al. (2022)^32^	3/60	3/60	OU: CWS, retinal vasculitis	OU: Intra-retinal fluid, subretinal fluid, increased macular thickness.	OU: Decreased retinal vascularity, bilateral capillary closure, decreased vascularity in the choroid	Retinal vasculitis	6 weeks	6/6	6/9	OU: Complete recovery
Parakh et al. (2023)^33^	6/60	6/60	OU: Bilateral disc hyperaemia, CME, Peripapillary areas of greying extensive VS in the mid-periphery, Perivascular subretinal infiltration	OU: NSD and CME and inner retinal hyperreflectivity. OD: Prominent BALAD.	Peripheral capillary non-perfusion areas (OS> OD)	Retinal vasculitis	10 months	6/6	6/60	OD: Complete recovery RE. OS: Blind.
Babu Kalpana et al. (2023)^34^	6/6	Counting fingers close to face	CWS, vitreous cells, disc pallor with NVD, IRH, macular ischemia with macular oedema	NA	Large areas of non-perfusion in the macula	Retinal vasculitis.	3 months	6/6	Counting fingers 2m	OU: No improvement.
Sharum et al. (2024)^35^	FC 1 feet	6/24	OD: Optic disc swollen, Dot-blot, flame-shaped IRH, pale retina, attenuated arteries, and venous tortuosities.	NA	OD: delayed filling of the central retinal artery, with complete retinal vein occlusion. OS: Normal	CRAO and CRVO secondary to occlusive vasculitis	4 months	PL	6/24	OU: No improvement
Sharum et al. (2024)^35^	6/36	6/36	OD: Pale optic disc Oedematous macula, Multiple CWS, Attenuation of retinal arteries. OS: Pink optic disc. Oedematous macula, Multiple CWS, deep IRH.	NA	OD: Large areas of capillary non-perfusion in both eyes with involvement of the macula in the right. OS: Hypo fluorescence area at the macula with poor arterial and venous filling.	OU: occlusive vasculitis involving artery and veins with right eye CRAO.	NA	6/24	6/9	Marked Improvement
Current (2025)	3/60	3/60	OU: Macular oedema, perifoveal hard exudates in fan-shaped pattern, multiple CWS. OS: Similar findings plus perifoveal flame-shaped bleed inferonasal to the fovea.	OD: serous detachment with intra-retinal fluid nasally and hyper-reflective spots. OS: Intra-retinal fluid all across and hyper-reflective spots.	NA	Purtscher retinopathy	8 months	6/9	6/9	Marked improvement
Current study (2025)	FC 1m	FC 1m	OU: Macular oedema, Extensive CWS, Few areas with RH	OU: serous retinal detachments (OD > OS)	NA	Retinal vasculitis	4 months	6/9	6/12	Marked improvement

BCVA: Best corrected visual acuity; BRAO: Branch retinal artery occlusion; BALAD: Bacillary layer detachment; CME: Cystoid macular oedema; CRS: Cherry red spot; CWS: Cotton-wool spots; CRAO: Central retinal artery occlusion; CRVO; Central retinal vein occlusion; FC; Finger counting; FFA; Fundus fluorescein angiography; HM: Hand Motion; IRH: Intraretinal haemorrhage; NA: Not available; NSD: Neurosensory detachment; NVD: Neovascularization of disc; NVR: Neovascularization of retina; NAION; Non-arteritic anterior ischemic optic neuropathy; OD: Oculus dexter; OS; Oculus sinister; OU; Oculus uterque; OCT: Optical coherence tomography; ODE: Optic disc oedema; PL: Perception of light; RD: Retinal detachment; RH: Retinal haemorrhage; RAO: Retinal artery occlusion; VD: venous dilatation; VH: Vitreous haemorrhage; VS: vascular sheathing.

## DISCUSSION

JLR can involve various ocular structures, including the lacrimal glands, orbits, conjunctival and scleral vessels, cornea, ciliary body, choroid, retina, and optic nerves.^10^ Retina is the second most common ocular structure involved after dry eyes, in JSLE.^2^ Retinal involvement can manifest as lupus retinopathy, which includes microangiopathy, retinal vasculitis, or vaso-occlusive retinopathy, all directly attributed to the inflammatory and occlusive nature of the disease. The other causes of retinal involvement, like neuroretinitis, systemic hypertension, and thrombocytopenia, are indirectly linked to the disease activity.^10^ The third form of retinopathy, caused by drug toxicity, particularly hydroxychloroquine, is a prime concern for clinicians.^3^ Retinal vasculitis secondary to intraocular infections like varicella-zoster has also been reported in JSLE patients.^14^

The pathogenesis of SLE retinopathy involves immune complex-mediated microangiopathic and microthrombosis due to endothelial inflammation, influenced by APLA and complement activation. It can be classified into three forms: microangiopathy, the most common and the mildest; vaso-occlusive retinopathy, the most severe; and retinal vasculitis, which precedes vaso-occlusive retinopathy. Retinal microangiopathy is characterised by retinal ischemia due to immune-complex deposition and presents with cotton-wool spots and minimal haemorrhages, while retinal vasculitis involves acute onset perivascular sheathing, leading to retinal ischemia, oedema, and haemorrhage, causing severe visual impairment. The development of vaso-occlusive retinopathy can lead to neovascularisation, subretinal haemorrhages, and eventually retinal detachment. Lupus retinopathy parallels the systemic disease activity, acting as an indicator of inflammation.^46^

This review on vision-threatening JLR analysed 27 cases in 25 articles, revealing a mean patient age of 13.7 years (SD ± 2.2 years), with a female-to-male ratio of 2:1. The median duration of SLE before developing lupus retinopathy was one month (range: 0 - 60 months) for 25 cases, with two cases missing this information. In our study, two female patients aged 17 years and 15 years developed retinopathy at 1.5 years and 2 months post-diagnosis, respectively. Thus, severe lupus retinopathy can develop both early as well as late in juvenile lupus which has also been seen in the case reports. Most cases (17, 63.0%), like ours, involved both eyes with vision loss, and treatment was initiated at a median of three days (range: 0 - 2 months) after vision loss in 19 cases (70.4%). Our patients presented relatively late at 2 weeks and one month after vision deterioration. Among the clinical domains, constitutional and mucocutaneous symptoms were the most common in 15 (55.6%) cases each. Cutaneous vasculitis was found in 3 cases (11.1%), highlighting a shared pathogenesis with lupus retinopathy. Neuropsychiatric lupus (NPLE) was diagnosed in five (18.5%) cases, while six (22.2%) had lupus nephritis. In the haematological domain, AIHA and thrombocytopenia were present in 3 (11.1%) and 5 (18.5%) cases, respectively. Both of our cases had proliferative lupus nephritis. Mucocutaneous involvement was common to both, while only one had cutaneous small vessel vasculitis. AIHA was an early presenting feature in the first case, and none had NPLE.

Data on autoantibodies against ENAs were available in 20 cases (74.1%). Anti-dsDNA was most commonly present in 16 (80.0%) of these cases. Anti-Ro and anti-RNP were present in eight (40.0%) and seven (35.0%) cases, respectively, followed by anti-Sm antibody in five (25.0%). Anti-Nuc and anti-Ribo P antibodies were the least commonly present in one case each (5.0%). In our case series, anti-Sm and anti-Nuc antibodies were present in both cases. Anti-dsDNA, anti-Hi, anti-RNP, anti-Ro, and anti-Ribo P were the other autoantibodies present in one case each. Serological activity data on serum C3 and/or C4 were available for 20 cases (74.1%), with 15 (75.0%) showing low levels of C3 and/or C4.

The data on APLA were present in 19 cases (70.4%), with six (31.6%) testing positive for APLA, and three (15.8%) were double positive, suggestive of a high-risk APLA profile.^47^ The low prevalence of APLA in JLR highlights the fact that thrombotic pathways are not the sole pathomechanism in JLR. Both our cases were APLA-negative and did not require antithrombotic agents.

The modalities of ocular imaging are depicted in Table 2. which highlights key findings: cotton-wool spots and intraretinal haemorrhages, which were the most prevalent in 18 (66.7%) and 16 (59.3%) cases, respectively. Retinal vessel abnormalities, including dilatation, narrowing, or obliteration, were found in 11 (40.7%) cases, while vascular sheathing was reported in six (22.2%) cases. Neovascularisation of the retina and disc occurred in 7.4% and 3.7% of cases, respectively. Macular oedema was present in 10 (37.0%), and cherry-red spots in 2 (7.4%) cases. Five (18.5%) developed vitreous haemorrhage, with limited vision improvement in some (11.1%) cases, and 3.7% developed tractional retinal detachment. The two cases in our case series had similar findings as the case reports in JLR. Our findings mirrored those in the JLR case reports, where cotton wool spots, intraretinal haemorrhages, and macular oedema were the most prevalent. The majority (17, 63%) of the JLR cases had retinal vasculitis, while only seven (25.9%) had vaso-occlusive retinopathy. Three of them (11.1%) had mixed patterns with combined features of vaso-occlusive retinopathy and retinal vasculitis. The patients in our case reports had retinal vasculitis, which was the predominant form of vision-threatening lupus retinopathy in juvenile age groups. FFA demonstrated filling defects in 16 case reports (59.3%). FFA was not performed in our case and OCTA images showed segmentation error due to extensive retinal detachments.

The treatment of these cases primarily focused on aggressive systemic immunosuppression, with high-dose glucocorticoids administered in 26 cases (96.3%). CYC was the most common steroid-sparing immunosuppressive agent used, in 12 cases (44.4%), followed by rituximab (RTX) and azathioprine (AZA) in seven (25.9%) cases each. MMF was used in four cases (14.8%), while tacrolimus (Tac) and methotrexate (MTX) were used in a single case (3.7%) each. Intravenous immunoglobulin (2; 7.4%) and plasma-pheresis (1; 3.7%) were the other treatment modalities rarely utilised in the case reports. Among the intraocular treatment modalities, retinal photocoagulation was most commonly used in 11 cases (40.74%), with intraretinal anti-vascular endothelial growth factor (anti-VEGF) and intravitreal glucocorticoids used in two (7.4%) cases each. Intravitreal tissue-plasminogen activator (t-PA), cryo-retinopexy, and pars plana vitrectomy were rarely utilised, in one case (3.7%) each.

Four out of the six APLA-positive JLR cases required systemic immunosuppression in addition to antithrombotics, with two cases ^23,31^ reporting remarkable vision improvement with immunosuppression alone. This shows that immunosuppression is the cornerstone for the treatment of APLA-positive JLR cases, with antithrombotics playing an adjunctive role. Only one of the four patients of APLA-positive JLR (25%), who received adjunctive antithrombotics, did not improve. Interestingly, the two vision-threatening JLR cases reported by Parchand et al.,^18^ and Guleria et al.,^26^ were APLA-negative but received adjunctive antithrombotic therapy, with mixed outcomes. These case reports bear testimony to the fact, that while APLA is significant in JLR pathogenesis,^46^ immune-complex vasculitis may play a more crucial role. HCQ known for its immunomodulatory and anti-thrombotic properties,^48^ has been used in one-third of the case reports (9; 33.3%) in the presence of JLR. The data on the addition of HCQ to the existing immunosuppressive regimen is not available in 17 cases, while in the only case reported by Alhassan et al,^29^ HCQ was withheld. Although the risk of HCQ-induced retinal toxicity is less than 1% in five years,^49^ the benefits should outweigh the risks, necessitating baseline screening within a year of initiation and annual follow-ups.^50^

In 24 of the 27 case reports, the median follow-up duration was 6 months (range: 1 week to 48 months). Seven cases (25.9%) either had no improvement or a deterioration of visual acuity. Three cases (11.1%), showed improvement in one eye only. However, the majority of the cases (63.0%) reported had a marked improvement or a near complete recovery of visual acuity. Two cases of JLR achieved nearly complete visual recovery after 8 months and 4 months with high-dose steroids and monthly IV CYC. One case is now on with MMF, while HCQ was continued throughout treatment. No antithrombotics were used, and only one case required IVTA; none required pan-retinal photocoagulation (PRP), intraretinal anti-VEGF therapy, intravitreal t-PA, cryo-retinopexy, or pars plana vitrectomy.

The present case-based review, elaborating on the existing cases of vision-threatening juvenile lupus retinopathy, is a novel attempt to highlight a serious complication of JSLE, considering the limited data in the literature on childhood-onset lupus retinopathy. However, our present case-based review had its limitations. One of the limitations was the inability to perform fundus fluorescein angiography for a better delineation of retinal vessels. A longer follow-up duration, required for better monitoring of visual acuity, was lacking in our case reports. The disease activity scores, like the SLEDAI, were not mentioned in the majority of the case reports. We also highlighted the coexistence of APLA and severe JLR, and suggest that this association needs to be addressed in a separate, adequately powered study.

## CONCLUSIONS AND FUTURE PERSPECTIVES

In conclusion, lupus retinopathy is one of the rare, yet severe manifestations of juvenile lupus, requiring prompt diagnosis and management. Key diagnostic tools include fundoscopy, optical coherence tomography, and fundus-fluorescein angiography. Aggressive systemic immunosuppression, with high-dose glucocorticoids and other steroid-sparing agents, is the cornerstone of treatment. However, intraocular administration of steroids or PRP is often required in severe refractory cases. The visual outcomes have been encouraging in the majority of cases. The authors believe that further exploration into the association of APLA with juvenile lupus retinopathy could be studied with a control group to better delineate the role of these antibodies in the pathogenesis of JLR. Longer follow-up of the cases might provide a better idea of the long-term outcome of this rare presentation of juvenile lupus.

## Data Availability

The data, images and results of statistical analyses can be available from the corresponding author on reasonable request.
